# Substance Abuse in Emerging Adults: The Role of Neuromelanin and Ventral Striatal Response to Social and Monetary Rewards

**DOI:** 10.3390/brainsci12030352

**Published:** 2022-03-04

**Authors:** Johanna M. Jarcho, James B. Wyngaarden, Camille R. Johnston, Megan Quarmley, David V. Smith, Clifford M. Cassidy

**Affiliations:** 1 Department of Psychology, Temple University, Philadelphia, PA 19122, USA; james.wyngaarden@temple.edu (J.B.W.); camille.johnston@temple.edu (C.R.J.); megan.quarmley@temple.edu (M.Q.); david.v.smith@temple.edu (D.V.S.); 2 University of Ottawa Institute of Mental Health Research, Affiliated with The Royal, Ottawa, ON K1Z 8N3, Canada; clifford.cassidy@theroyal.ca

**Keywords:** adolescent SUD, risk factors, fMRI, NM-MRI, peer feedback, midbrain

## Abstract

Perturbations in dopamine system function may increase risk of substance use disorder (SUD). We recently demonstrated that neuromelanin (NM) MRI signal in the substantia nigra, a non-invasive index of dopamine system function, is elevated in long term cocaine users (Cassidy et al., 2020). However, it is unclear whether elevated NM-MRI signal is linked to risk of SUD, or is a byproduct of long-term drug use. Our prior work failed to show relations between NM-MRI signal and functional engagement of ventral striatum during a monetary reward task. However, social experiences are commonly linked to drug use and relapse. Given that, NM-MRI signal may be more closely linked to ventral striatal engagement during social, rather than monetary reward processing. Emerging adults (*n* = 33, 21.88 ± 4.35 years) with varying levels of substance abuse, but without SUD, underwent NM-MRI and fMRI during social and monetary reward processing tasks. Voxelwise analysis within the substantia nigra (SN) demonstrated lower NM-MRI signal was associated with more severe substance abuse. Lower right ventral striatal engagement to social reward was also associated with more severe substance abuse. This relation was moderated by SN NM-MRI signal such that diminished striatal response to reward was associated with greater substance abuse among those with low NM-MRI signal, but lower substance abuse among those with high NM-MRI signal. Unexpectedly, higher right ventral striatal engagement during monetary reward was associated with more severe substance abuse. This relation was moderated by SN NM-MRI signal such that greater striatal response to reward was associated with greater substance abuse among those with low NM-MRI signal. Taken together, we provide preliminary evidence that, in emerging adults, low rather than high dopamine system function may increase risk of substance abuse, and strengthen the association between substance use and the brain’s sensitivity to social and monetary outcomes in different ways.

## 1. Introduction

Substance use disorders among late adolescents and emerging adults are common, costly, and on the rise [1,2]. Since 2016, there has been a 61% increase in substance use among 8th graders [3]. Given the fact that substance use typically peaks between 18−25 years of age [4], we are likely to see a substantial increase in substance abuse and substance use disorders (SUDs) among late adolescents and emerging adults over the next several years. Yet, only a minority of those who try drugs of abuse develop a SUD [5]. Hence, attempts have been made to identify risk factors for SUD. Given its role in reward processing, perturbations in dopamine system function has been one target of investigation. However, most measures of dopamine system function are invasive, and require the use of radioactive tracers. Thus, it can be difficult to directly test for perturbations in younger populations. Less is known about how perturbations in dopamine system function may interact with psychosocial events that influence substance abuse. For instance, among late adolescents and emerging adults, substance use often occurs in a social context [6,7,8], and social stressors commonly precipitate binging and relapse [9]. Using the relatively novel and non-invasive technique of neuromelanin sensitive-MRI (NM-MRI), previously shown to correlate with dopamine system function [10], we sought to test the extent to which dopamine system function relates to substance abuse behavior in emerging adults without SUD. Moreover, we aimed to test whether differences in dopamine system function moderate the relation between neural response to social feedback, measured using fMRI, and substance abuse.

Alterations of dopamine system function have been identified in individuals with numerous SUDs [11,12]. These studies have largely relied on data acquired using positron emission tomography (PET). As PET imaging entails exposure to radiation, it is contraindicated for most research in younger at-risk populations. Therefore, much of what we know about dopamine system function and substance abuse in humans comes from adult populations. However, onset of substance abuse and development of SUD typically occurs in late adolescence and early adulthood [5]. As substance abuse itself affects dopamine system functioning, it is difficult to disentangle whether dopamine system perturbations in most studies of SUD reflect the cause or consequence of disordered behavior. Hence, it is critical to adopt non-invasive techniques for measuring dopamine system function in the population at greatest risk of development SUD—emerging adults.

Non-invasive NM-MRI has recently been shown to provide a putative proxy measure of dopamine function [10,13]. Neuromelanin is a pigment generated from the oxidation of cytosolic dopamine. It accumulates gradually over the lifespan in dopamine neurons of the substantia nigra (SN) [14]. As NM is bound to iron, it forms paramagnetic complexes that can be imaged using MRI [13,15,16]. NM-MRI has also been directly linked to dopamine release in the striatum [10]. NM-MRI can be used to quantify NM depletion in Parkinson’s disease [13,14,17], but can also detect altered dopamine system function in non-neurodegenerative disorders, such as schizophrenia [10,15]. We recently demonstrated that long-term cocaine users (M ± SD age = 47.3 ± 8.1 years; years of use = 21.9 ± 9.3 years) showed greater NM-MRI signal compared to non-users (age = 45.1 ± 10.2 years; [16]). One interpretation of these results is that SUD is associated with hyper-dopamine function and excess NM accumulation. However, it is unclear whether this increased signal reflected pre-existing risk of SUD (i.e., related to a cause of SUD), or increased accumulation due to long-term cocaine use (i.e., related to a consequence). Although many theories suggest that risk is related to hyperdopaminergic function [17,18,19], an alternative perspective is that hypodopaminergic function is associated with compensatory reward-seeking behavior, which promotes risk of SUD [20,21]. Thus, our results may reflect consequences (perhaps due to cocaine-linked alterations in dopamine trafficking, see [16]) rather than causes. To test whether NM-MRI signal relates to pre-existing risk of SUD, it is therefore critical to test its association with substance abuse in emerging adults who have not yet developed SUD.

Peer feedback becomes increasingly salient during adolescence [22,23,24], just as substance abuse begins to rise [5]. Among adolescents and young adults, substance abuse most often occurs in a social context [6,8], and peer victimization or rejection commonly precipitate consumption [25]. Thus, it is possible that dopamine system function may moderate the effect of social experiences on substance abuse. Some direct evidence for this comes from studies in non-human primates. Specifically, the introduction of a social context (i.e., group housing following isolation) provokes a relatively rapid increase in D2 dopamine receptor density, but only among monkeys who rise to the top of the social hierarchy. This change in dopamine system function in turn buffers against the reinforcing effects of cocaine. Conversely, monkeys who become subordinate show no change in dopamine system function, and become more vulnerable to the reinforcing effects of cocaine [26]. Thus, hypodopaminergic function coupled with a lack of positive social experiences may increase risk of substance abuse.

In humans, one way to test how peer-based experiences “get under the skin” is to measure functional brain response to peer feedback. Indeed, fMRI studies demonstrate that activity in the ventral striatum, a major hub of the dopaminergic reward-processing system, is more responsive to peer feedback among adolescents compared to children [27,28,29]. Despite the preponderance of evidence that social experiences impact substance use in late adolescence and early adulthood, few studies have tested the link between adolescent neural response to social feedback and substance abuse [30,31,32]. A more consistent pattern emerges in the monetary domain; specifically, substance abuse is associated with reduced striatal responding to monetary reward (e.g., [17,33,34,35,36,37], but see [38,39,40]). However, results are mixed when it comes to *risk* of substance abuse (e.g., [41,42,43,44]). Some studies show increased risk of substance abuse is associated with hyper-ventral striatal engagement to reward, whereas others show the opposite relation or null effects.

These inconsistencies may be partially due to variability in experimental design within and across studies. Few experiments use well-controlled methods to directly test brain response to social and monetary rewards. Indeed, our recent work in adolescents and emerging adults demonstrates differences in neural response across social and monetary domains [45,46,47,48], and finds that symptoms of psychopathology specifically relate to neural response to social, but not monetary reward [47,48]. Given the specific impact of social experiences on substance abuse, it is therefore possible that neural response to social rewards may uniquely relate to risk of substance abuse. Although diminished dopamine function may increase risk of substance abuse, it is plausible that it may also potentiate hyper- or hypo-responsivity to social reward. For example, greater sensitivity to peer feedback coupled with diminished dopamine function could increase sensitivity to peer pressure, thereby promoting substance abuse via a young adult’s need to belong. Alternatively, blunted sensitivity to peer feedback coupled with diminished dopamine function could promote substance abuse motivated by the failure of social experiences to elicit appetitive experiences.

This study had two goals. The first goal was to test the relation between substance abuse and NM-MRI signal in emerging adults without SUD. We hypothesize that, consistent with some theories that hypodopaminergic function promotes risk of SUD, low NM-MRI signal will be associated with more severe substance abuse. The second goal was to test the extent to which NM-MRI signal moderated the relation between ventral striatal response to reward and substance abuse within social and monetary domains. Given the importance of peers during emerging adulthood and that social experiences commonly predict substance use, we hypothesize that NM-MRI signal will moderate the relation between (hyper- or hypo-reactive) ventral striatal response to reward and substance abuse, specifically in the social, but not monetary domain. 

## 2. Materials and Methods

### 2.1. Participants

Participants (*n* = 33; females = 21) were undergraduates (*M* = 21.88 ± 4.35 years) recruited from Stony Brook University who received course credit for their participation. Participants were free of psychotropic medication and had no contraindications for fMRI. Additional participants were excluded from analyses due to poor quality fMRI data (*n* = 1), and for having high substance abuse problem scores (*n* = 2; outliers > 2.5 SD from mean). Informed written consent was obtained prior to participation, and all procedures were approved by the Institutional Review Board at Stony Brook University and follow the principles of the Declaration of Helsinki. 

### 2.2. Substance Use

Substance use was measured via self-report on the substance abuse subscale (α = 0.94) of the Externalizing Spectrum Inventory-brief form (ESI-bf; [49]). The substance abuse subscale is a sum of responses (on a 0 = false to 3 = true scale) to 49 questions about use of, and problems resulting from use of, marijuana, non-marijuana substances (e.g., cocaine, opioids), and alcohol. A subscale for substance abuse problems was a sum of the subset of 27 problem-related questions (α = 0.76).

### 2.3. Procedure

Prior to the experimental session, participants were told they were completing a social evaluation study and were asked to submit a digital picture of themselves that would be sent to other purported participants their age across the country. Participants believed that these peers would receive a text message asking them to view the photo and indicate whether they thought they would ‘‘like’’ the participant. The picture would then disappear after 5 min. At the beginning of the experimental session, participants were told that they would be asked to predict which peers ‘‘liked’’ them and that they would also complete a monetary prediction task. Participants underwent fMRI while completing the monetary and social tasks. Structural and NM-MRIs were also acquired. At the end of the session, participants responded to questions about their experience with the task to ensure they were engaged and believed the credibility of the peer feedback. Participants were then debriefed. Although a subset of participants were not deceived (*n* = 15), they did not differ from deceived participants in terms of age, substance use, NM-MRI signal, or ventral striatal response to social or monetary reward, and were therefore included in analyses to retain statistical power.

### 2.4. fMRI-Based Tasks

The monetary and social reward tasks were administered using E-Prime software (Psychology Software Tools Inc., 2016, (E-Prime 2.0). Retrieved from http://www.pstnet.com, last accessed on 10 January 2018) and presented in a counterbalanced order. There were four conditions (monetary win, monetary loss, social like, and social dislike) that were presented in a counterbalanced, randomized order. Each condition included 30 trials. Each task was completed across two 4.55 min runs. Each run included two blocks: one block of monetary win or social like trials, and one block of monetary loss or social dislike trials (15 trials per block). Trials were separated by a variable duration intertrial interval (1100–11,600 ms; *M* = 3500 ms). For this report, we describe results from the positively valenced blocks because they are conceptually similar to typical reward processing tasks in which accurate choices are associated with positive outcomes. We did not have specific hypotheses about the relation between neuromelanin and brain function during outcomes in the negatively valenced blocks. See other reports for results related to neural responses during negative outcomes (e.g., [46,47,48]). 

#### 2.4.1. Monetary Reward Task 

**Figure 1:**
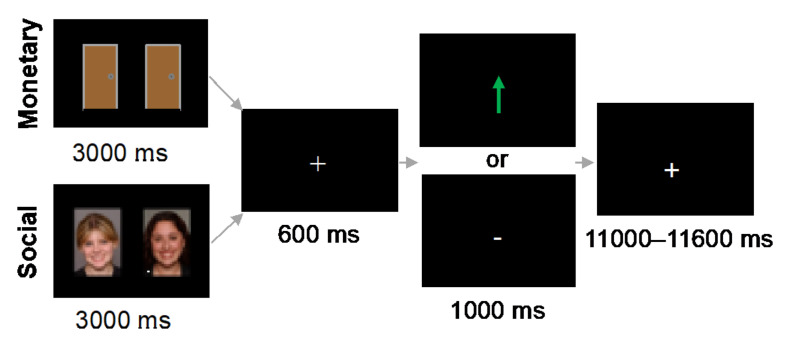
fMRI-based monetary and social reward tasks.

For the monetary reward task (Figure 1), participants were instructed to choose the door behind which there was a USD 0.25 prize. Participants were told that there were three possible scenarios for each trial: (1) both doors contained a USD 0.25 monetary win; (2) one door contained a USD 0.25 monetary win while the other door resulted in a break-even outcome; or (3) both doors resulted in a break-even outcome. This ensured that the feedback the participant received would only be informative about the door they chose and not the door they did not choose. Each trial began with the presentation of two identical doors (3000 ms). Participants then used a button box to choose either the left or right door. After stimulus offset, a fixation cross was presented for 600 ms before participants received feedback (1000 ms). Feedback was either a green arrow pointing upward (↑) meaning the participant chose the monetary win door (rewarding feedback), or a white horizontal dash (-), which indicated the participant chose the break-even door, resulting in no monetary win (non-rewarding feedback).

#### 2.4.2. Social Reward Task 

The social reward task was identical to the monetary task except pictures of gender-matched pairs of peers (i.e., two female faces or two male faces) were presented instead of doors (Figure 1). Participants were instructed to choose the peer that liked the participant, based on the photo that the participant provided. The social reward task consisted of 60 images of age-matched peers compiled from multiple sources (National Institute of Mental Health’s Child Emotional Faces picture set [50] and internet databases of non-copyrighted images). The pictures of purported peers had positive facial expressions, were cropped so that individuals were pictured from their shoulders up and were edited to have an identical solid gray background. Images were constrained to a standard size (2.75-inch width × 4-inch height). Participants were told that there were three possible scenarios for each trial: (1) both people said they would like the participant; (2) one person said they would like the participant while the other person never rated the participant; or (3) neither person rated the participant. Each trial began with the presentation of two peers (3000 ms). Participants then used a button box to choose either the left or right peer. After stimulus offset, a fixation cross was presented for 600 ms before participants received feedback (1000 ms). Feedback was either a green arrow pointing upward (↑) meaning the participant chose the peer who liked them (rewarding feedback), or a white horizontal dash (-), which indicated the participant chose the peer who never rated them, resulting in no positive social feedback (non-rewarding feedback). 

### 2.5. MRI Acquisition 

All images were acquired using a 3T Siemens PRISMA MRI scanner (Siemens AG, Muenchen, Germany) with a 20 channel coil.

#### 2.5.1. Structural MRI

To facilitate anatomical localization and coregistration of functional and NM-MRI data, a high-resolution structural scan was acquired (sagittal plane) with a T1-weighted magnetization-prepared rapid acquisition gradient echo (MPRAGE) sequence (250 mm in FOV, TR = 1900 ms, TE = 2.53 ms, voxel size of 1.0 × 1.0 × 1.0 mm^3^, flip angle = 9°).

#### 2.5.2. NM-MRI

NM sensitive MRI images were acquired using a 2D gradient response echo sequence with magnetization transfer contrast (220 mm in FOV, 10 slices, TR = 273 ms, TE = 3.87 ms, voxel size of 0.4 × 0.4 × 3.0 mm, flip angle = 40°, interleaved slice acquisition, magnetization transfer frequency offset = 1200 Hz; acquisition time = 11.02 min). The 10-slice-prescription protocol consisted of orienting the image stack along the anterior-commissure–posterior-commissure line and placing the top slice 3 mm below the floor of the third ventricle, viewed on a sagittal plane in the middle of the brain (see [10], for more detail). This protocol provided coverage of SN-containing portions of the midbrain. 

#### 2.5.3. fMRI

Blood Oxygenation Level-Dependent (BOLD) sensitive functional images were acquired using a gradient echo-planar imaging (EPI) sequence (224 mm in FOV, 37 slices, TR = 2100 ms, TE = 23 ms, voxel size of 2.3 × 2.3 × 3.5 mm, flip angle = 83°, interleaved slice acquisition). Each run included 130 functional volumes. 

## 3. Data Analysis

### 3.1. NM-MRI 

#### 3.1.1. Preprocessing

SPM12 was used to co-register NM-MRI scans to T1-weighted scans and DARTEL routines were used to normalize data to MNI space. Unsmoothed, normalized NM-MRI data were resampled to 1 mm isotropic. Intensity normalization and spatial smoothing were then performed using custom Matlab scripts (see [10] for further preprocessing details).

#### 3.1.2. Individual Level Analysis

**Figure 2:**
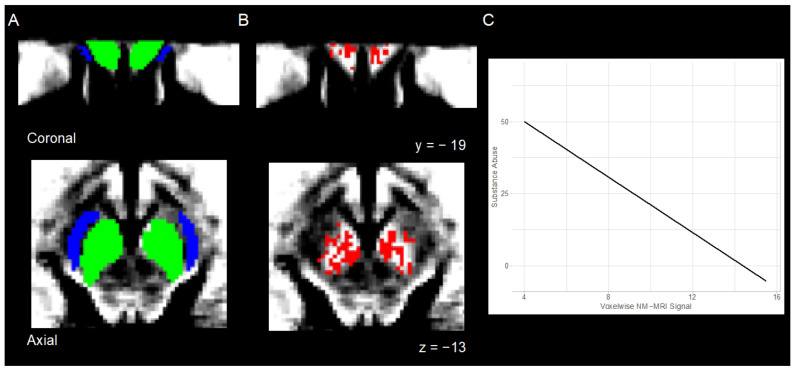
Template NM-MRI images were created by averaging the spatially normalized NM-MRI images from all participants. Overlays reflect (**A**) anatomical substantia nigra (green) and crus cerebri (blue) masks; (**B**) results from voxelwise analysis showing a significant negative relation between substance abuse and NM-MRI signal in substantia nigra (red). (**C**) Scatter plot depicting this negative relation.

Analyses required the use of two anatomical masks generated using average spatially normalized NM-MRI images from previously studied participants ([10]; Figure 2A; see below). Given its high NM content, the substantia nigra (1807 resampled voxels) served as the region of interest (ROI), while the crus cerebri, white-matter tracts with minimal NM content, served as a reference region. Contrast-to-noise ratio (CNR) for each participant and voxel within the SN (v) was calculated as the relative change in NM-MRI signal intensity (I) from the reference region (RR): *CNR_V_* = (*I_V_* − *mode*(*I_RR_*))/*mode*(*I_RR_*). The mode (*I_RR_*) was calculated for each participant from kernel-smoothing-function fit of a histogram of all voxels in the mask. 

### 3.2. fMRI 

#### 3.2.1. Preprocessing

Preprocessing and fMRI analyses were conducted using AFNI (Cox, 1996). Standard preprocessing steps were implemented with afni_proc.py; this included slice timing, coregistration, smoothing to 6 mm full-width half maximum (FWHM) to a Gaussian kernel, spatial normalizing to standard Talairach space, and resampling, which resulted in 2-mm^3^ voxels. To account for large movements, temporally adjacent repetition times (TRs) with a Euclidean-norm motion derivative greater than 1 mm were omitted from the model via censoring. No participant had more than 1% of TRs omitted for any given event type.

#### 3.2.2. Individual Level Analysis

Task-specific events (spanning the duration of each event) were modeled using a block function. An additional six regressors modeled motion residuals. Based on a priori hypotheses, we performed an ROI analysis on the ventral striatum, as defined by the Harvard Oxford Atlas [51,52,53]. Anatomical masks of bilateral ventral striatum were transformed from MNI to Talairach space using AFNI’s 3dWarp tool. Partial voxels were removed and masks were eroded by 1 level using AFNI’s 3dmask_tool. The resulting left (97 resampled voxels) and right (90 resampled voxels) ventral striatum masks are depicted in Supplementary Figure S1. For each ROI, signal change data for rewarding vs. non-rewarding feedback for each domain (monetary: win–break-even; social: like–did not rate) was then extracted for each subject. 

### 3.3. Group Level Analyses

All statistical analyses were performed R’s ‘stats’ package (1.3.1.073 [54]). To achieve each of our experimental goals, we conducted two sets of analyses. 

#### 3.3.1. Relation between NM-MRI Signal and Substance Abuse 

First, a voxel-wise analysis within the SN mask was performed to determine whether substance abuse was correlated with NM-MRI signal in a significant number of voxels within the SN. These analyses were carried out in Matlab using custom scripts. Robust linear regression analyses were performed across participants for every voxel *v* within the SN mask, as (Equation (1)):

CNRv = β0 + β1·substance abuse (1)

The spatial extent threshold of an effect was defined as the number of voxels *k* (adjacent or nonadjacent) exhibiting a significant relationship between substance abuse and CNR (voxel-level height threshold for t-test of regression coefficient β1 of *p* < 0.05). Separate one-sided analyses tested for positive and negative relations. Permutation testing was performed to correct for multiple comparisons by determining whether an effect’s spatial extent *k* was greater than would be expected by chance (P_corrected_ < 0.05; 10,000 permutations, equivalent to a cluster-level familywise error-corrected *p* value). On each iteration, the order of substance abuse variable was randomly permuted across participants prior to performing voxelwise regression analysis. This provided a measure of spatial extent for each of 10,000 permuted datasets, forming a null distribution against which to calculate the probability of observing the spatial extent *k* of the effect in the true data by chance (P_corrected_). In this case, the voxel extent threshold was set to 255 voxels. Significant effects were plotted for visualization purposes and corresponding statistics are reported for completeness.

#### 3.3.2. Moderating Effect of NM-MRI Signal on the Relation between Ventral Striatal Function during Reward Processing and Substance Abuse 

For subsequent analyses, participant NM-MRI signal reflects a signal extracted from the anatomically defined SN. Specifically, the NM-MRI signal for each participant was calculated via Matlab as the average CNR across all voxels within the substantia nigra mask. Using this measure, we used PROCESS 3.5.3 [55] to test whether NM-MRI signal moderated the relation between ventral striatal response to social (like–did not rate) or monetary (win–break-even) rewards and substance abuse. For each analysis, the independent variable (X) was neural response to reward, the predictor variable (W) was average NM-MRI signal, and the dependent variable (Y) was substance abuse. Significant effects were probed using the Johnson–Neyman technique. Age may impact the salience of reward outcomes differently in social and monetary domains in emerging adults [27,28,29], thus models with and without age were compared. As including age did not impact overall results, age had low predictive value, and the goodness of fit (Mean Squared Error) was better for models without compared to with age, all results were derived from models that omitted age.

## 4. Results

### 4.1. Substance Abuse

Substance abuse scores were normally distributed (skewness = 0.77, SE = 0.41; kurtosis = −0.38; SE = 0.80). Average substance abuse score was 19.55 (*SD* = 19.69; range = 0–67), which is consistent with prior college samples (*M*~24) but much lower than clinical substance use disorder populations (*M*~84 [56]). Moreover, the majority of participants (64%) reported zero substance use problems, and had overall problem scores much lower (*M* = 1.48, *SD* = 2.71; range = 0–10) than clinical populations (*M*~42). Thus, this sample facilitated our goal of studying individuals at risk of, but without expression of, substance abuse disorder. 

### 4.2. Relation between NM-MRI Signal and Substance Abuse

Voxelwise analysis revealed substance abuse was negatively correlated with NM-MRI signal in a significant number of voxels within the SN (Figure 2B; 416 of 1807 SN voxels, P_corrected_ < 0.007). Figure 2C depicts this negative relation, which supported our hypothesis that individuals with lower intensity NM-MRI signal would engage in greater substance abuse behavior (adjusted *R*^2^ = 0.439, *F*(1,31) = 26.032, *b* = −4.82, *SE* = 0.94, *t*(32) = −5.10, *p* < 0.001).

### 4.3. Moderating Effect of NM-MRI Signal on the Relation between Ventral Striatal Function during Reward Processing and Substance Abuse 

#### 4.3.1. Social Reward 

**Figure 3:**
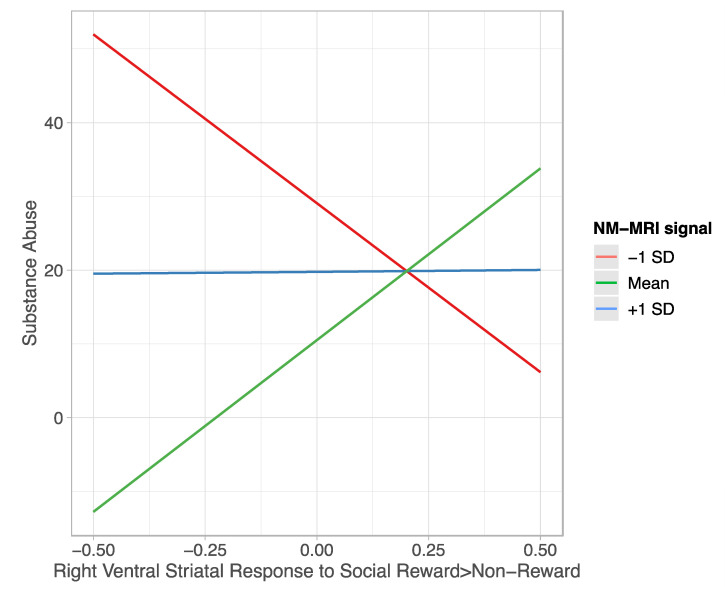
Depiction of moderating effect of NM-MRI signal on the relation between right ventral striatal response to social reward (vs. non-reward) and substance abuse. SD = standard deviation.

Regarding the right ventral striatum, the overall model was significant; *R*^2^ = 0.266, *F*(3, 29) = 3.493, and *p* = 0.0281. Consistent with our hypothesis and voxelwise analyses, a main effect of NM-MRI signal emerged (*b* =  −4.472, 95% C.I., (−7.639, −1.304), *t*(32) = −2.888, and *p* = *0*.00073), such that a lower NM-MRI signal was associated with more severe substance abuse. A similar relation emerged for neural response to social reward (*b* = −186.228, 95% C.I., (−355.012, −17.445), *t*(32) = −2.247, and *p* = *0*.032), such that weaker striatal response to social reward was associated with more severe substance abuse. These main effects were qualified by an interaction. Consistent with our hypothesis, NM-MRI signal moderated the relationship between neural response to social reward and severity of substance abuse (Δ*R^2^* = 0.132, *F*(1,29) = 5.224; *b*  =  22.256, 95% C.I. (3.025, 41.487), and *p* = *0*.025; Figure 3). Johnson–Neyman analyses suggest an inverse relation between striatal response to reward and substance abuse depending on NM-MRI signal. Specifically, diminished striatal response to reward was associated with greater substance abuse behavior among those with low NM-MRI signal (<4.45). Conversely, diminished striatal response to reward was associated with lower substance abuse behavior among those with high NM-MRI signal (>11.24). Finally, there was no relation between striatal response to reward and substance abuse in those with moderate levels of NM-MRI signal. 

Regarding the left ventral striatum, the overall model was not significant; *R*^2^ = 0.156 *F*(3, 29) = 1.780, and *p* = 0.173. Consistent with our hypothesis and voxelwise analyses, a main effect of NM-MRI signal emerged (*b* =  −3.76, 95% C.I., (−7.253, −0.267), *t*(32) = −2.202, and *p* = 0.036), such that lower NM-MRI signal was associated with more severe substance abuse. There was no main effect of neural response to social reward (*b* = −69.945, 95% C.I., −312.934, 173.045), *t*(32) = −0.589, and *p* = 0.561), nor was there social reward by NM-MRI signal interaction (Δ*R*^2^ = 0.013 *F*(1,29) = 0.454; *b* = 9.903, 95% C.I., (−20.157, 39.963), and *p* = 0.506).

#### 4.3.2. Monetary Reward 

**Figure 4:**
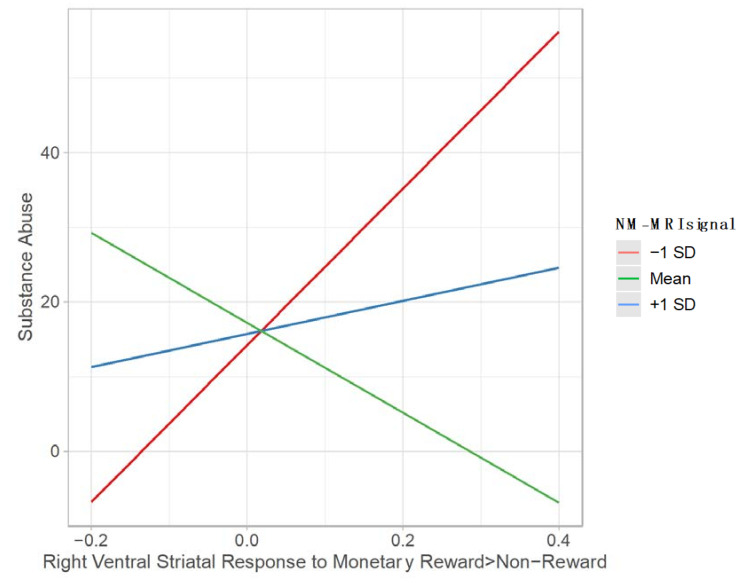
Depiction of moderating effect of NM-MRI signal on the relation between right ventral striatal response to monetary reward (vs. non-reward) and substance abuse. SD = standard deviation.

Regarding the right ventral striatum, the overall model was significant; *R*^2^ = 0.306, *F*(3, 29) = 4.262, and *p* = 0.0131. There were no main effects of NM-MRI signal (*b* = 0.725, 95% C.I., (−3.798, 5.248), *t*(32) = 0.328, and *p* = 0.745). However, there was a main effect of neural response to monetary reward (*b* = 356.254, 95% C.I., (75.929, 636,579), *t*(32) = −2.599, and *p* = 0.0145), such that greater striatal response to monetary reward was associated with more severe substance abuse. This main effect was qualified by an interaction. Inconsistent with our hypotheses, NM-MRI signal moderated the relationship between neural response to social reward and severity of substance abuse (Δ*R*^2^ = 0.145, *F*(1,29) = 6.049; *b*  =  −39.820, 95% C.I. (−72.935, −6.706), and *p* = 0.021; Figure 4). Johnson–Neyman analyses suggest greater striatal response to reward was associated with greater substance abuse behavior among those with low NM-MRI signal (<7.37). There was no relation between striatal response to reward and substance abuse in those with high or moderate levels of NM-MRI signal. 

Regarding the left ventral striatum, the overall model was not significant; *R*^2^ = 0.197 *F*(3, 29) = 2.376, and *p* = 0.091. There were no main effects of NM-MRI signal (*b*  =  −2.230, 95% C.I., (−6.182, 1.722), *t*(32) = −1.154, and *p* = 0.258) or neural response to monetary reward (*b*  =  127.729, 95% C.I., (−72.402, 327.859), *t*(32) = 1.305, and *p* = 0.202), nor was there a monetary reward by NM-MRI signal interaction (Δ*R*^2^ = 0.055, *F*(1,29) = 1.985; *b*  =  −15.248, 95% C.I., (−37.382, 6.887), and *p* = 0.170).

## 5. Discussion

In this study, we tested the relation between NM-MRI signal, substance abuse, and ventral striatal sensitivity to social and monetary reward in emerging adults. Three sets of encouraging preliminary results emerge. First, lower NM-MRI signal in the substantia nigra, a proxy measure of dopamine system functioning, is associated with greater substance abuse behaviors. Second, among those with hypo-striatal response to positive social feedback, high NM-MRI signal may be protective against, while low NM-MRI signal may increase the risk of, substance abuse behavior. Third, hyper-striatal response to positive monetary feedback coupled with low NM-MRI signal may increase risk of substance abuse behaviors. Although results require replication with a larger sample, taken together, these data: (1) provide initial support for the hypothesis that hypodopamineric function increases risk of SUD; (2) suggest prior work demonstrating positive relations between NM-MRI signal and SUD may reflect long-term consequences of sustained use rather than risk; and (3) help shed light on mechanisms by which sensitivity to social and non-social rewards may contribute to substance abuse and relapse.

This is the first report we are aware of to test the relation between NM-MRI signal and substance abuse in individuals without a long-term SUD. Unlike our prior results, which demonstrated that long-term cocaine users in their 40s had greater NM-MRI signal compared to non-users [16], we demonstrate that substance abuse in emerging adults is associated with lower NM-MRI signal. This is consistent with hypotheses that hypodopaminergic function increases risk of SUD [20,21]. The fact that the same pattern of results emerged across both voxelwise and ROI-based analyses bolsters confidence for the present results and suggests our prior findings of increased NM accumulation may be the consequence of long-term cocaine use. Individual differences in NM-MRI signal is not likely the consequence of substance use in the present sample, given that NM accumulation is a slow and gradual process [13,57], and that our sample has been using substances for a relatively short time, with few substance-related problems. Despite this, longitudinal research that measures NM-MRI signal prior to the onset of substance use is needed to definitively confirm that lower NM-MRI signal predicts subsequent use and development of SUD. Nevertheless, the present cross-sectional results provide compelling preliminary data that demonstrate the utility of using NM-MRI signal to test dopamine system perturbations in younger populations and support the promise of NM-MRI to measure substance abuse risk. 

There is a strong drive for peer acceptance during adolescence and early adulthood, and social experiences often prompt substance use in this population. Yet, few studies have tested the relation between neural response to social rewards and substance abuse in emerging adults. Here, we found that diminished right ventral striatal response to social reward is associated greater substance abuse behavior. While initially this effect may seem contradictory, it is possible that the failure to benefit from positive peer experiences may motivate substance abuse. For example, non-human primate studies show that failure to upregulate dopamine system function in response to social experiences increases cocaine use [26]. Moreover, hyperstriatal responding to positive social feedback is normative during adolescence and emerging adulthood [58]. Individuals who fail to adequately engage such systems during positive social encounters may therefore seek appetitive experiences through other avenues, such as substance abuse.

We also found that NM-MRI signal moderates the relation between right ventral striatal response to social reward and substance abuse behaviors. Specifically, low NM-MRI may potentiate the relation between diminished striatal response and substance abuse. This supports the idea that hypodopaminergic tone, in conjunction with a failure to trigger appetitive responses to positive peer experiences, may lead to more risky reward-seeking behavior. However, high NM-MRI signal seems to buffer young adults who are hypo-responsive to positive social feedback. One reason for this may be that higher dopaminergic tone is protective against the failure to experience positive social outcomes as appetitive. Likewise, greater response to positive social feedback seems to eliminate the differential effects of NM-MRI signal on substance abuse. Thus, if normative neural responses to peer feedback are engaged, more moderate levels of substance abuse occur. This suggests a socio-emotional mechanism that protects against substance abuse. 

The majority of studies to test the link between neural response to reward and substance abuse have focused on the monetary domain. Here, we found that greater right ventral striatal response to winning money is associated with greater substance abuse behavior. This is consistent with hypotheses suggesting that risk of SUD is associated with heightened, rather than diminished reward circuit function [17,18,19]. Yet, inconsistent with these theories, low rather than high NM-MRI signal potentiates the relation between brain function and substance abuse behaviors. This provides some support for the idea that hypodopaminergic tone in conjunction with an exaggerated appetitive response to positive non-social experiences may lead to more risky reward-seeking behavior. Thus, hypo- rather than hyper-dopaminergic function seems to potentiate risk of substance abuse depending on right ventral striatal response to social and monetary reward outcomes. The fact that potentiating effects are related to hypostriatal responding for social reward and hyperstriatal responding in the monetary reward requires further inquiry, and suggests that outcome domain is a critical feature that should be considered in future work on risk of SUD.

Despite its strengths, this study is not without its limitations. The biggest limitation is the relatively small sample size. This is particularly important in light of recent work that suggests fMRI studies assessing individual differences with relatively small samples should be interpreted with caution [59]. Likewise, while our prior work using a variant of this task indicates that it has strong reliability/dependability (based on measures of split-half-reliability, Cronbach’s alpha, and G theory dependability measures) [45], its modest number of trials may compound this issue. Likewise, the limited sample size precluded our capacity to test for potential sex differences. This is critical given the link between sex differences in risk of SUD and corresponding development of neural circuits implicated in affective processing [60]. Relatedly, the composition of the sample had some drawbacks. Namely, the majority of participants in this sample were female—a limitation, given these sex differences. Moreover, while a subset of participants did not believe they received feedback from actual peers during the fMRI task, deceived and non-deceived participants did not differ on any characteristics measured in the study, including striatal response to feedback. This is not entirely surprising given that numerous studies that ask participants to imagine that feedback is real (i.e., do not use deception), still elicit robust neural response to that feedback (e.g., [61,62,63,64]). Additional studies with larger samples that use more robust deception techniques are clearly needed to replicate and extend the current findings. Second, while we demonstrate a relation between blunted striatal response to positive social feedback and substance abuse, and greater striatal response to positive monetary feedback and substance abuse, we cannot claim that brain response reflects diminished or enhanced affective experiences. Future work that relates brain function to affective responses are needed to confirm this relation. Furthermore, additional work is generally needed to quantify, and thus equalize, the value ascribed to reward outcomes across domains. For instance, given the salience of peers during adolescence, a larger monetary win may be required to yield an equivalent value reward as positive peer feedback. Only when methods are devised for determining subjective equivalence for monetary and social value can domain-specific effects truly be isolated. Third, this study used cross-sectional methods. Although we have provided preliminary evidence for the relation between striatal response to social and monetary reward, NM-MRI signal, and risk of SUD, longitudinal research is the only way to determine if these relations predict increased substance abuse or conversion to SUD. Finally, the sample was not selected based on individual differences in reward sensitivity. We have addressed this issue in ongoing research with similar measures in which we specifically study emerging adults with a range of reward sensitivity who are at risk of, but do not meet criteria for SUD [65].

While more work is clearly warranted, the present results are an important first step in demonstrating the link between dopamine system function and substance abuse in emerging adults. Moreover, we provide insight into a potential mechanism that could help explain the common association between social experiences and substance abuse in late adolescents and emerging adults, while drawing attention to the need for a nuanced approach to considering reward domain in future research. Taken together, these results make an important initial contribution to our understanding of the causes and consequences of substance abuse and may guide the development of clinical interventions that focus on responses to social and non-social feedback in an efforts to curb substance abuse in young adults.

## Data Availability

Data are available upon request to the corresponding author.

## Supplementary Materials

The following supporting information can be downloaded at: https://www.mdpi.com/article/10.3390/brainsci12030352/s1, Figure S1: Anatomical masks of left and right ventral striatum.
