# Supplementary figures and images for: Substance Abuse in Emerging Adults: The Role of Neuromelanin and Ventral Striatal Response to Social and Monetary Rewards
Source: Brain Sci. 2022 Mar 4;12(3):352. doi: 10.3390/brainsci12030352 (PMC8946041; doi:10.3390/brainsci12030352)

Supplementary Materials:

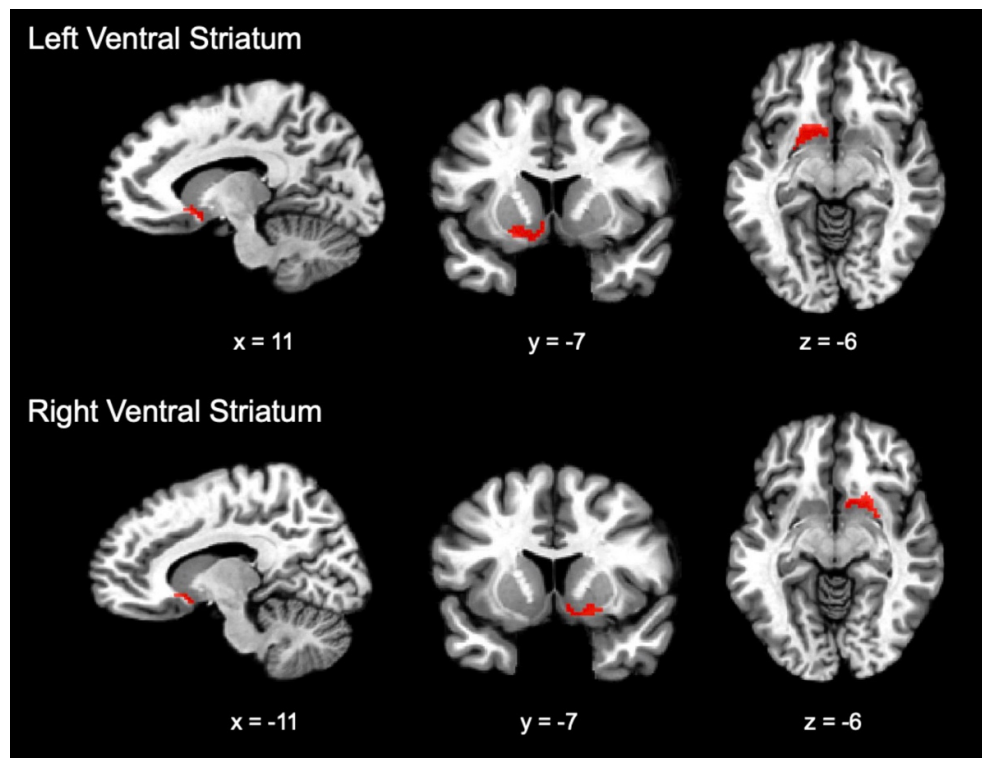

**Figure S1:** Anatomical masks of left and right ventral striatum.

Supplement: Supplementary file 1 [file brainsci-12-00352-s001.zip › brainsci-1531921-supplementary.pdf]
